# The genome sequence of the Marbled White Spot, *Protodeltote pygarga *(Hufnagel, 1766)

**DOI:** 10.12688/wellcomeopenres.20571.1

**Published:** 2024-01-08

**Authors:** Douglas Boyes, Owen T. Lewis, University of Oxford and Wytham Woods Genome Acquisition Lab, Darwin Tree of Life Barcoding collective, Wellcome Sanger Institute Tree of Life Management, Samples and Laboratory team, Wellcome Sanger Institute Scientific Operations: Sequencing Operations, Wellcome Sanger Institute Tree of Life Core Informatics team, Tree of Life Core Informatics collective, Darwin Tree of Life Consortium

**Affiliations:** 1 UK Centre for Ecology & Hydrology, Wallingford, England, UK; 2 University of Oxford, Oxford, England, UK

**Keywords:** Protodeltote pygarga, Marbled White Spot, genome sequence, chromosomal, Lepidoptera

## Abstract

We present a genome assembly from an individual male *Protodeltote pygarga* (the Marbled White Spot; Arthropoda; Insecta; Lepidoptera; Noctuidae). The genome sequence is 421.1 megabases in span. Most of the assembly is scaffolded into 31 chromosomal pseudomolecules, including the Z sex chromosome. The mitochondrial genome has also been assembled and is 15.48 kilobases in length. Gene annotation of this assembly on Ensembl identified 17,784 protein coding genes.

## Species taxonomy

Eukaryota; Metazoa; Eumetazoa; Bilateria; Protostomia; Ecdysozoa; Panarthropoda; Arthropoda; Mandibulata; Pancrustacea; Hexapoda; Insecta; Dicondylia; Pterygota; Neoptera; Endopterygota; Amphiesmenoptera; Lepidoptera; Glossata; Neolepidoptera; Heteroneura; Ditrysia; Obtectomera; Noctuoidea; Noctuidae; Eustrotiinae; *Protodeltote*; *Protodeltote pygarga* (Hufnagel, 1766) (NCBI:txid708063).

## Background

The Marbled White Spot ( *Protodeltote pygarga*) is a small noctuid moth. This species has undergone a marked range expansion in Britain and Ireland in recent decades. It now occurs widely in grassland, heathland, woodland and moorland habitats, but is absent from Scotland and northern England ( Randle *et al.*, 2019). Its distribution extends across much of Europe to western and central Asia, and it is also recorded from Japan, Korea and China ( GBIF Secretariat, 2023).

The larval host plants are grasses. This species has a single annual generation and overwinters as a pupa underground ( Waring *et al.*, 2017).

The genome of the marbled white spot, *Protodeltote pygarga*, was sequenced as part of the Darwin Tree of Life Project, a collaborative effort to sequence all named eukaryotic species in the Atlantic Archipelago of Britain and Ireland. Here we present a chromosomally complete genome sequence for *Protodeltote pygarga*, based on one male specimen from Wytham Woods, Oxfordshire.

## Genome sequence report

**Figure 1.**
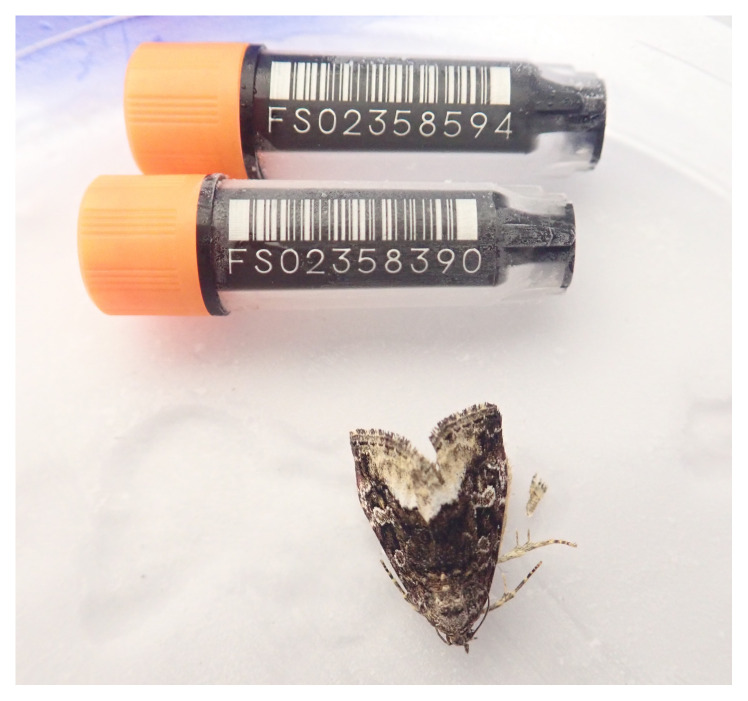
Photograph of the *Protodeltote pygarga* (ilProPyga1) specimen used for genome sequencing.

The genome was sequenced from one male *Protodeltote pygarga* ( Figure 1) collected from Wytham Woods, Oxfordshire, UK (51.77, –1.34). A total of 92-fold coverage in Pacific Biosciences single-molecule HiFi long reads was generated. Primary assembly contigs were scaffolded with chromosome conformation Hi-C data. Manual assembly curation corrected 7 missing joins or mis-joins and removed 2 haplotypic duplications, reducing the assembly length by 0.45%.

**Figure 2.**
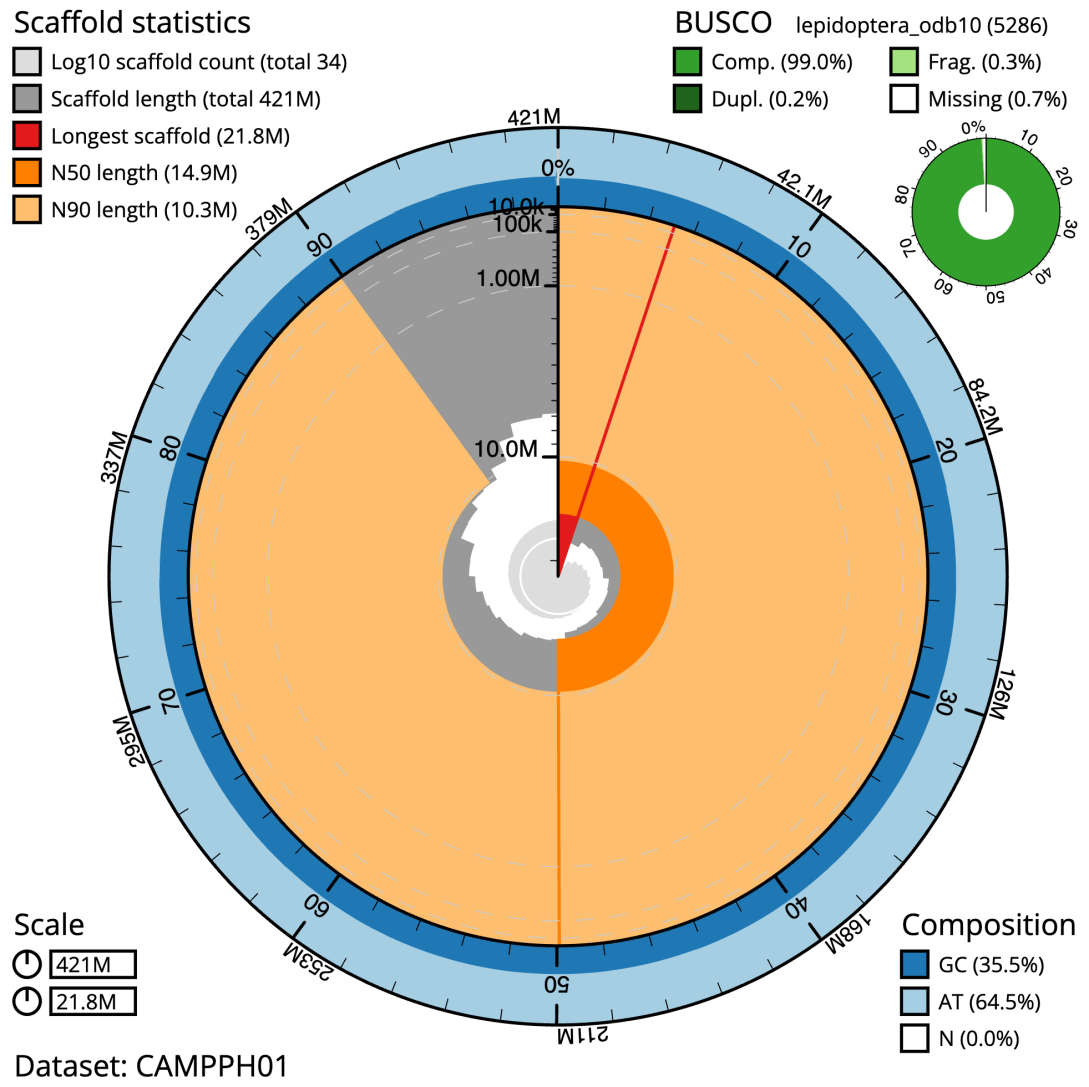
Genome assembly of *Protodeltote pygarga*, ilProPyga1.2: metrics. The BlobToolKit Snailplot shows N50 metrics and BUSCO gene completeness. The main plot is divided into 1,000 size-ordered bins around the circumference with each bin representing 0.1% of the 421,153,487 bp assembly. The distribution of scaffold lengths is shown in dark grey with the plot radius scaled to the longest scaffold present in the assembly (21,803,012 bp, shown in red). Orange and pale-orange arcs show the N50 and N90 scaffold lengths (14,901,961 and 10,302,340 bp), respectively. The pale grey spiral shows the cumulative scaffold count on a log scale with white scale lines showing successive orders of magnitude. The blue and pale-blue area around the outside of the plot shows the distribution of GC, AT and N percentages in the same bins as the inner plot. A summary of complete, fragmented, duplicated and missing BUSCO genes in the lepidoptera_odb10 set is shown in the top right. An interactive version of this figure is available at https://blobtoolkit.genomehubs.org/view/Protodeltote%20pygarga/dataset/CAMPPH01/snail.

**Figure 3.**
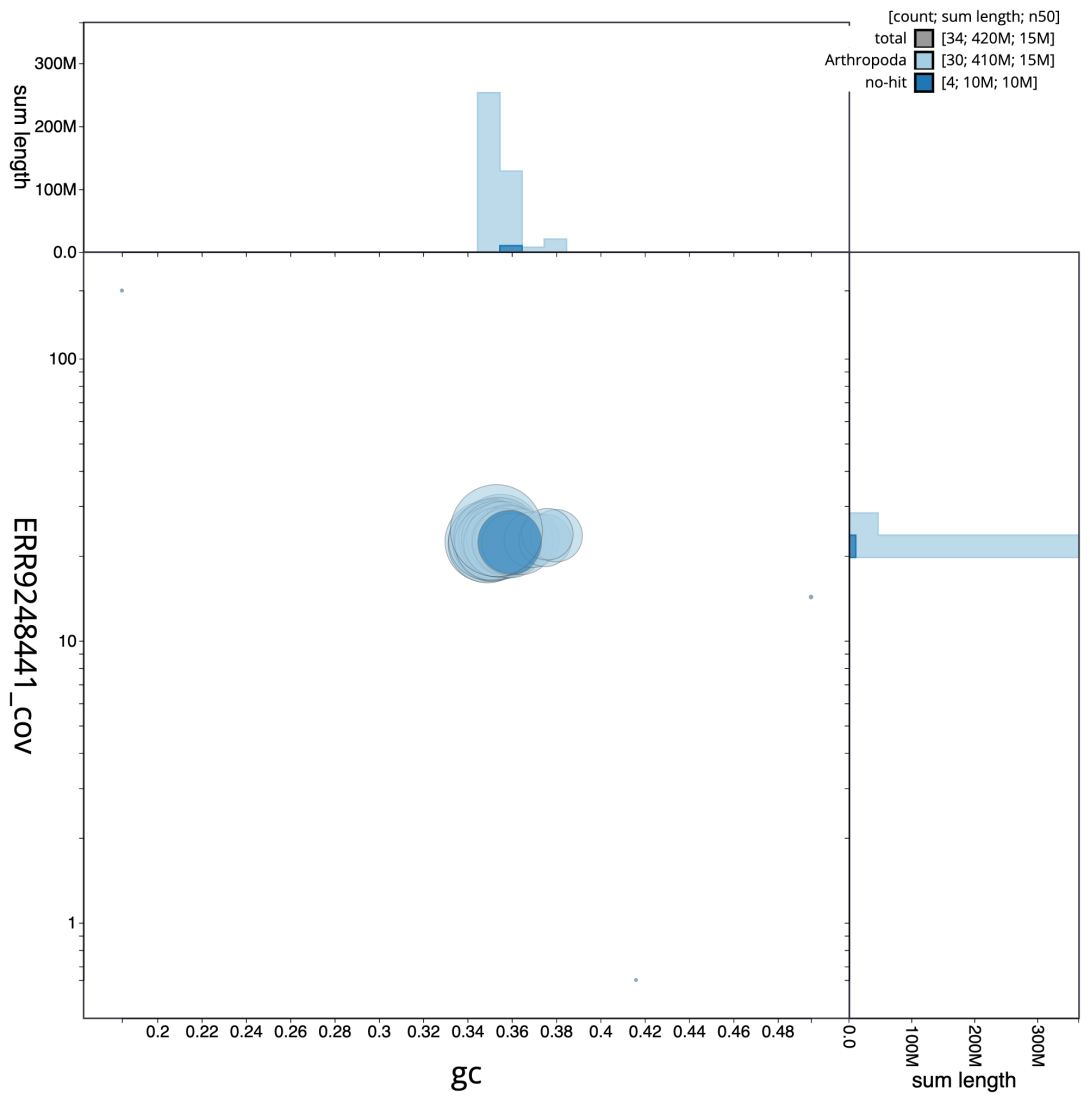
Genome assembly of *Protodeltote pygarga*, ilProPyga1.2: BlobToolKit GC-coverage plot. Scaffolds are coloured by phylum. Circles are sized in proportion to sequence length. Histograms show the distribution of scaffold length sum along each axis. An interactive version of this figure is available at https://blobtoolkit.genomehubs.org/view/Protodeltote%20pygarga/dataset/CAMPPH01/blob.

**Figure 4.**
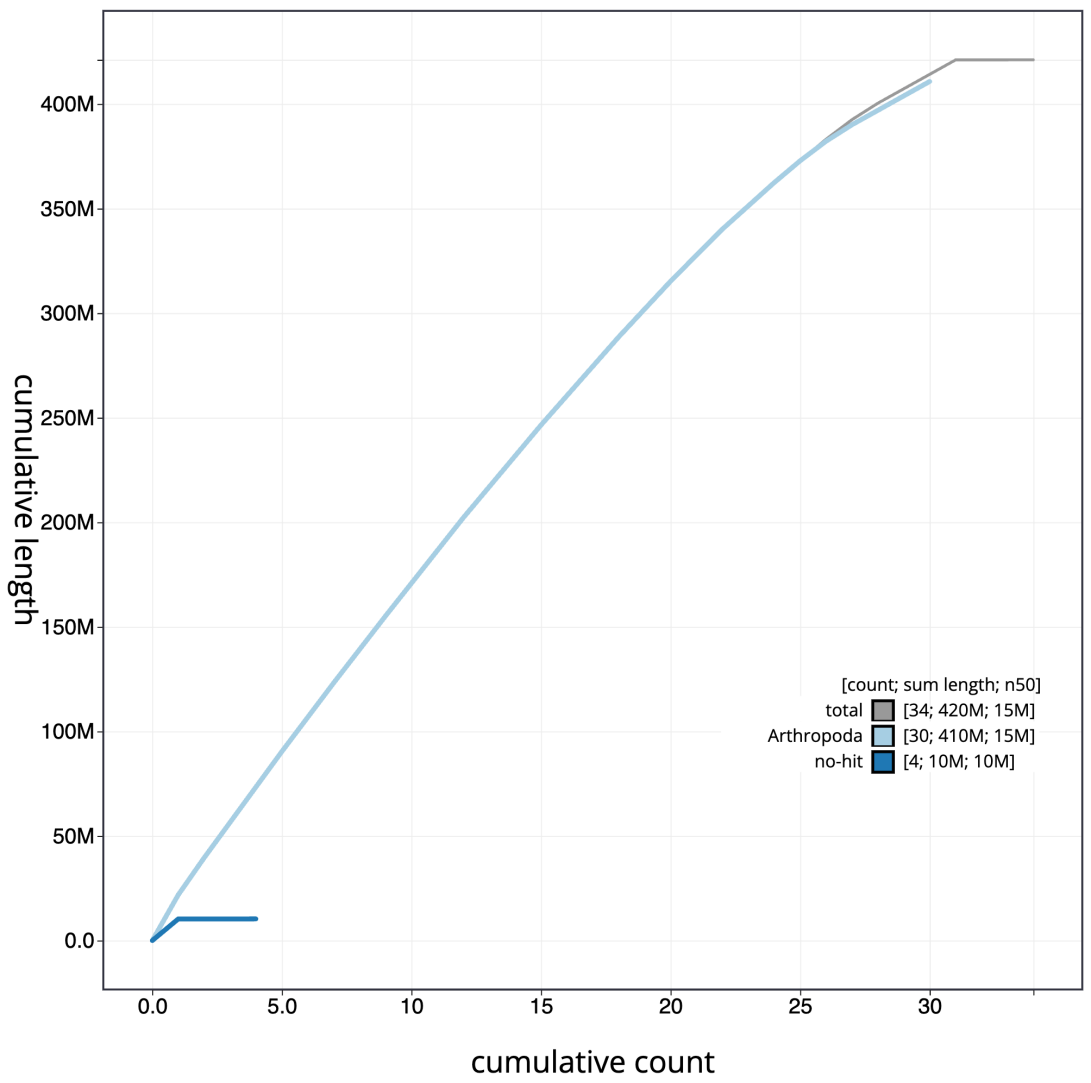
Genome assembly of *Protodeltote pygarga*, ilProPyga1.2: BlobToolKit cumulative sequence plot. The grey line shows cumulative length for all scaffolds. Coloured lines show cumulative lengths of scaffolds assigned to each phylum using the buscogenes taxrule. An interactive version of this figure is available at https://blobtoolkit.genomehubs.org/view/Protodeltote%20pygarga/dataset/CAMPPH01/cumulative.

**Figure 5.**
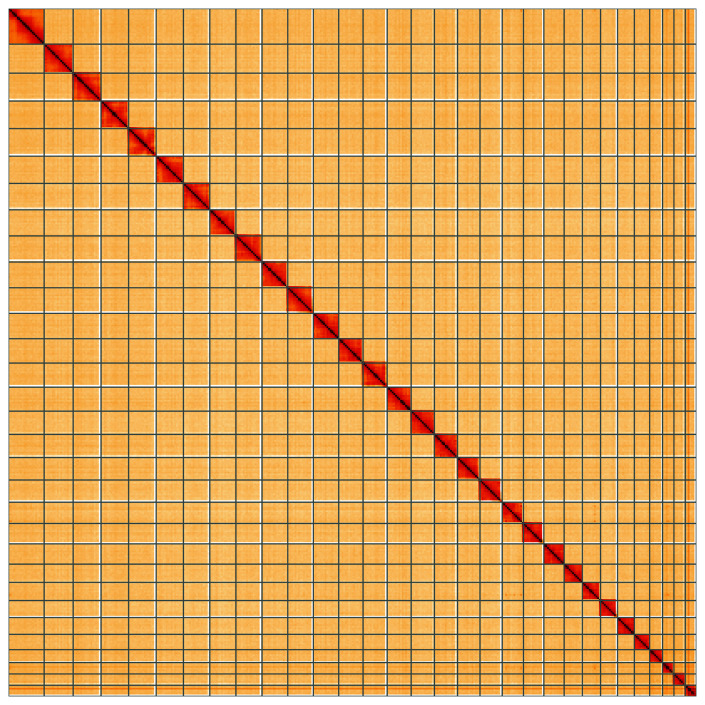
Genome assembly of *Protodeltote pygarga*, ilProPyga1.2: Hi-C contact map of the ilProPyga1.2 assembly, visualised using HiGlass. Chromosomes are shown in order of size from left to right and top to bottom. An interactive version of this figure may be viewed at https://genome-note-higlass.tol.sanger.ac.uk/l/?d=NRR27qs_Twah2X0btFEQTQ.

The final assembly has a total length of 421.1 Mb in 33 sequence scaffolds with a scaffold N50 of 14.9 Mb ( Table 1). The snailplot in Figure 2 provides a summary of the assembly statistics, while the distribution of reads on GC proportion and coverage is shown in Figure 3. The cumulative assembly plot in Figure 4 shows curves for subsets of scaffolds assigned to different phyla. Most (99.98 %) of the assembly sequence was assigned to 31 chromosomal-level scaffolds, representing 30 autosomes and the Z sex chromosome. Chromosome-scale scaffolds confirmed by the Hi-C data are named in order of size ( Figure 5; Table 2). While not fully phased, the assembly deposited is of one haplotype. Contigs corresponding to the second haplotype have also been deposited. The mitochondrial genome was also assembled and can be found as a contig within the multifasta file of the genome submission.

**Table 1.** Genome data for *Protodeltote pygarga*, ilProPyga1.2.

Project accession data		
Assembly identifier	ilProPyga1.2	
Species	*Protodeltote pygarga*	
Specimen	ilProPyga1	
NCBI taxonomy ID	708063	
BioProject	PRJEB51452	
BioSample ID	SAMEA7631560	
Isolate information	ilProPyga1, male: thorax (Hi-C data)	
Assembly metrics *		*Benchmark*
Consensus quality (QV)	69.4	*≥ 50*
*k*-mer completeness	100%	*≥ 95%*
BUSCO **	C:99.0%[S:98.8%,D:0.2%], F:0.3%,M:0.7%,n:5,286	*C ≥ 95%*
Percentage of assembly mapped to chromosomes	99.98%	*≥ 95%*
Sex chromosomes	Z chromosome	*localised homologous pairs*
Organelles	Mitochondrial genome assembled	*complete single alleles*
Raw data accessions		
PacificBiosciences SEQUEL II	ERR9284042, ERR9284043	
Hi-C Illumina	ERR9248439	
Genome assembly		
Assembly accession	GCA_936450705.2	
*Accession of alternate haplotype*	GCA_936440985.2	
Span (Mb)	421.1	
Number of contigs	44	
Contig N50 length (Mb)	14.7	
Number of scaffolds	33	
Scaffold N50 length (Mb)	14.9	
Longest scaffold (Mb)	21.8	
Genome annotation		
Number of protein-coding genes	17,784	
Number of gene transcripts	17,968	

* Assembly metric benchmarks are adapted from column VGP-2020 of “Table 1: Proposed standards and metrics for defining genome assembly quality” from ( Rhie *et al.*, 2021). ** BUSCO scores based on the lepidoptera_odb10 BUSCO set using v5.3.2. C = complete [S = single copy, D = duplicated], F = fragmented, M = missing, n = number of orthologues in comparison. A full set of BUSCO scores is available at https://blobtoolkit.genomehubs.org/view/Protodeltote%20pygarga/dataset/CAMPPH01/busco.

**Table 2.** Chromosomal pseudomolecules in the genome assembly of *Protodeltote pygarga*, ilProPyga1.

INSDC accession	Chromosome	Length (Mb)	GC%
OW388249.2	1	17.74	35.0
OW388250.2	2	17.08	35.5
OW388251.2	3	16.72	35.0
OW388252.2	4	16.89	35.5
OW388253.2	5	16.86	35.5
OW388254.2	6	16.11	35.0
OW388255.2	7	15.99	35.0
OW388256.2	8	16.0	35.0
OW388257.2	9	15.73	35.0
OW388258.2	10	15.69	35.5
OW388259.2	11	15.53	35.0
OW388260.2	12	14.9	35.0
OW388261.2	13	14.78	35.5
OW388262.2	14	14.66	35.5
OW388263.2	15	14.25	35.0
OW388264.2	16	14.14	35.5
OW388265.2	17	13.78	35.5
OW388266.2	18	13.58	36.0
OW388267.2	19	13.02	36.0
OW388268.2	20	12.48	35.5
OW388269.2	21	12.43	36.0
OW388270.2	22	11.16	35.5
OW388271.2	23	11.11	36.5
OW388272.2	24	10.43	36.0
OW388273.2	25	10.3	36.0
OW388274.2	26	9.37	36.0
OW388275.2	27	7.87	37.0
OW388276.2	28	6.97	37.5
OW388277.2	29	6.98	38.0
OW388278.2	30	6.76	37.5
OW388248.2	Z	21.8	35.5
OW388279.2	MT	0.02	18.5

The estimated Quality Value (QV) of the final assembly is 69.4 with *k*-mer completeness of 100%, and the assembly has a BUSCO v5.3.2 completeness of 99.0% (single = 98.8%, duplicated = 0.2%), using the lepidoptera_odb10 reference set ( *n* = 5,286).

Metadata for specimens, barcode results, spectra estimates, sequencing runs, contaminants and pre-curation assembly statistics are given at https://links.tol.sanger.ac.uk/species/708063.

## Genome annotation report

The *Protodeltote pygarga* genome assembly (GCA_936450705.2) was annotated using the Ensembl rapid annotation pipeline ( Table 1; https://rapid.ensembl.org/Protodeltote_pygarga_GCA_936450705.2/Info/Index). The resulting annotation includes 17,784 transcribed mRNAs from 17,968 protein-coding genes. 

## Methods

### Sample acquisition and nucleic acid extraction

A male *Protodeltote pygarga* (specimen ID Ox000464, ToLID ilProPyga1) was collected from Wytham Woods, Oxfordshire (biological vice-county Berkshire), UK (latitude 51.77, longitude –1.34) on 2020-06-13, using a light trap. The specimen was collected and identified by Douglas Boyes (University of Oxford) and preserved on dry ice.

High molecular weight (HMW) DNA was extracted at the Tree of Life laboratory, Wellcome Sanger Institute (WSI), following a sequence of core procedures: sample preparation; sample homogenisation; HMW DNA extraction; DNA fragmentation; and DNA clean-up. In sample preparation, the ilProPyga1 sample was weighed and dissected on dry ice ( Jay *et al*., 2023). Tissue from the abdomen was homogenised using a PowerMasher II tissue disruptor ( Denton *et al.*, 2023a). HMW DNA was extracted using the Automated MagAttract v1 protocol ( Sheerin *et al.*, 2023). DNA was sheared into an average fragment size of 12–20 kb in a Megaruptor 3 system with speed setting 30 ( Todorovic *et al.*, 2023). Sheared DNA was purified by solid-phase reversible immobilisation ( Strickland *et al.*, 2023): in brief, the method employs a 1.8X ratio of AMPure PB beads to sample to eliminate shorter fragments and concentrate the DNA. The concentration of the sheared and purified DNA was assessed using a Nanodrop spectrophotometer and Qubit Fluorometer and Qubit dsDNA High Sensitivity Assay kit. Fragment size distribution was evaluated by running the sample on the FemtoPulse system.

Protocols developed by the Wellcome Sanger Institute (WSI) Tree of Life core laboratory have been deposited on protocols.io ( Denton *et al.*, 2023b).

### Sequencing

Pacific Biosciences HiFi circular consensus DNA sequencing libraries were constructed according to the manufacturers’ instructions. DNA sequencing was performed by the Scientific Operations core at the WSI on Pacific Biosciences SEQUEL II (HiFi) and Illumina NovaSeq 6000 (10X) instruments. Hi-C data were also generated from thorax tissue of ilProPyga1 using the Arima2 kit and sequenced on the Illumina NovaSeq 6000 instrument.

### Genome assembly, curation and evaluation

Assembly was carried out with Hifiasm ( Cheng *et al.*, 2021) and haplotypic duplication was identified and removed with purge_dups ( Guan *et al.*, 2020). The assembly was then scaffolded with Hi-C data ( Rao *et al.*, 2014) using YaHS ( Zhou *et al.*, 2023). The assembly was checked for contamination and corrected as described previously ( Howe *et al.*, 2021). Manual curation was performed using HiGlass ( Kerpedjiev *et al.*, 2018) and Pretext ( Harry, 2022). The mitochondrial genome was assembled using MitoHiFi ( Uliano-Silva *et al.*, 2023), which runs MitoFinder ( Allio *et al.*, 2020) or MITOS ( Bernt *et al.*, 2013) and uses these annotations to select the final mitochondrial contig and to ensure the general quality of the sequence.

A Hi-C map for the final assembly was produced using bwa-mem2 ( Vasimuddin *et al.*, 2019) in the Cooler file format ( Abdennur & Mirny, 2020). To assess the assembly metrics, the *k*-mer completeness and QV consensus quality values were calculated in Merqury ( Rhie *et al.*, 2020). This work was done using Nextflow ( Di Tommaso *et al.*, 2017) DSL2 pipelines “sanger-tol/readmapping” ( Surana *et al.*, 2023a) and “sanger-tol/genomenote” ( Surana *et al.*, 2023b). The genome was analysed within the BlobToolKit environment ( Challis *et al.*, 2020) and BUSCO scores ( Manni *et al.*, 2021; Simão *et al.*, 2015) were calculated.

Table 3 contains a list of relevant software tool versions and sources.

**Table 3.** Software tools: versions and sources.

Software tool	Version	Source
BlobToolKit	4.0.7	https://github.com/blobtoolkit/blobtoolkit
BUSCO	5.3.2	https://gitlab.com/ezlab/busco
Hifiasm	0.16.1-r375	https://github.com/chhylp123/hifiasm
HiGlass	1.11.6	https://github.com/higlass/higlass
Merqury	MerquryFK	https://github.com/thegenemyers/MERQURY.FK
MitoHiFi	2	https://github.com/marcelauliano/MitoHiFi
PretextView	0.2	https://github.com/wtsi-hpag/PretextView
purge_dups	1.2.3	https://github.com/dfguan/purge_dups
sanger-tol/genomenote	v1.0	https://github.com/sanger-tol/genomenote
sanger-tol/readmapping	1.1.0	https://github.com/sanger-tol/readmapping/tree/1.1.0
YaHS	yahs-1.1.91eebc2	https://github.com/c-zhou/yahs

### Genome annotation

The BRAKER2 pipeline ( Brůna *et al.*, 2021) was used in the default protein mode to generate annotation for the *Protodeltote pygarga* assembly (GCA_936450705.2) in Ensembl Rapid Release.

### Wellcome Sanger Institute – Legal and Governance

The materials that have contributed to this genome note have been supplied by a Darwin Tree of Life Partner. The submission of materials by a Darwin Tree of Life Partner is subject to the **‘Darwin Tree of Life Project Sampling Code of Practice’**, which can be found in full on the Darwin Tree of Life website here. By agreeing with and signing up to the Sampling Code of Practice, the Darwin Tree of Life Partner agrees they will meet the legal and ethical requirements and standards set out within this document in respect of all samples acquired for, and supplied to, the Darwin Tree of Life Project. 

Further, the Wellcome Sanger Institute employs a process whereby due diligence is carried out proportionate to the nature of the materials themselves, and the circumstances under which they have been/are to be collected and provided for use. The purpose of this is to address and mitigate any potential legal and/or ethical implications of receipt and use of the materials as part of the research project, and to ensure that in doing so we align with best practice wherever possible. The overarching areas of consideration are:

• Ethical review of provenance and sourcing of the material

• Legality of collection, transfer and use (national and international) 

Each transfer of samples is further undertaken according to a Research Collaboration Agreement or Material Transfer Agreement entered into by the Darwin Tree of Life Partner, Genome Research Limited (operating as the Wellcome Sanger Institute), and in some circumstances other Darwin Tree of Life collaborators.

## Data Availability

European Nucleotide Archive: *Protodeltote pygarga* (marbled white spot). Accession number PRJEB51452; https://identifiers.org/ena.embl/PRJEB51452 ( Wellcome Sanger Institute, 2022). The genome sequence is released openly for reuse. The *Protodeltote pygarga* genome sequencing initiative is part of the Darwin Tree of Life (DToL) project. All raw sequence data and the assembly have been deposited in INSDC databases. Raw data and assembly accession identifiers are reported in Table 1.

## Author information

Members of the University of Oxford and Wytham Woods Genome Acquisition Lab are listed here: https://doi.org/10.5281/zenodo.7125292.

Members of the Darwin Tree of Life Barcoding collective are listed here: https://doi.org/10.5281/zenodo.4893703.

Members of the Wellcome Sanger Institute Tree of Life Management, Samples and Laboratory team are listed here: https://doi.org/10.5281/zenodo.10066175.

Members of Wellcome Sanger Institute Scientific Operations: Sequencing Operations are listed here: https://doi.org/10.5281/zenodo.10043364.

Members of the Wellcome Sanger Institute Tree of Life Core Informatics team are listed here: https://doi.org/10.5281/zenodo.10066637.

Members of the Tree of Life Core Informatics collective are listed here: https://doi.org/10.5281/zenodo.5013541.

Members of the Darwin Tree of Life Consortium are listed here: https://doi.org/10.5281/zenodo.4783558.
