# Supplementary material for: The genome sequence of the Marbled White Spot, Protodeltote pygarga (Hufnagel, 1766)
Source: Wellcome Open Res. 2024 Jan 8;9:7. [Version 1] doi: 10.12688/wellcomeopenres.20571.1 (PMC11167331; doi:10.12688/wellcomeopenres.20571.1)
Supplement: Peer review history

# Reviewer report: Reviewer response for version 1

**DOI:** 10.21956/wellcomeopenres.22770.r78591

**Published:** 2024-08-01

**Authors:** Tyler Scott Alioto

**Affiliations:** 1 Fundacion Centro Nacional de Analisi Genomico, Barcelona, Catalonia, Spain

The genome note submitted by Boyes *et al.* reports the genome sequence of the Marbled White Spot, Proto Deltore pygarga. P. pygarga is a small moth that has increased its range across southern Britain and Ireland. It is distributed widely in Europe and Asia as well. It has a single annual generation, pupating underground over winter and the larval host plants are grasses. The male specimen sequenced was collected from Wytham Woods. 92-fold coverage of HiFi reads were generated and assembled with Hifiasm, run through purge_dups, scaffolded with 141 Gb (335x) of Arima2 Hi-C data using YaHS, and manually curated with few interventions necessary. The assembly is chromosome-scale and of high quality, meeting the minimum standards recommended by the Earth Biogenome Project. All protocols are appropriate and well-documented. All data conforms with FAIR principles, with read data and assemblies being available in the ENA. Blob toolkit figures are interactive. Additional QC data are provided at https://tolqc.cog.sanger.ac.uk/, supplementing the figures published in the data note.

 No major comments.

Minor comments:

1. A ZZ-male was collected and sequenced. I believe it is usually standard practice to try to sequence the heterogametic sex. The authors should provide an explanation for why they chose a ZZ male. Were there difficulties in collecting females? Are ZW females too hard to assemble? It would help the rest of the reference genome sequencing community to know the rationale for choices made or be made aware of difficulties encountered.
2. A general comment on the genome-note engine. I would like to see the Table 1 comparison to the now-old VGP standards "Assembly metric benchmarks are adapted from column VGP-2020 of “Table 1: Proposed standards and metrics for defining genome assembly quality” from (Rhie et al., 2021).” changed to a comparison to the latest EBP metric standards. Especially for non-vertebrate genomes.
3. Is there a way to indicate what protein set was used to annotate the genome with BRAKER2? In order to reproduce the annotation, this information would seem necessary. Please find a way to include such info (from Ensembl rapid) in the genome note template.

Are sufficient details of methods and materials provided to allow replication by others?

Yes

Is the rationale for creating the dataset(s) clearly described?

Yes

Are the datasets clearly presented in a useable and accessible format?

Yes

Are the protocols appropriate and is the work technically sound?

Yes

Reviewer Expertise:

genomics, genome assembly, gene prediction, annotation

I confirm that I have read this submission and believe that I have an appropriate level of expertise to confirm that it is of an acceptable scientific standard.

# Reviewer report: Reviewer response for version 1

**DOI:** 10.21956/wellcomeopenres.22770.r78595

**Published:** 2024-06-11

**Authors:** Maria Nilsson

**Affiliations:** 1 Senckenberg Biodiversity and Climate Research Centre, Frankfurt am Main, Germany

The genome of the Marbled White Spot ( *Protodeltote pygarga*) has been sequenced from a single male individual captured using a light trap. The Marbled White Spot is a commonly occurring moth that is found throughout Europe and has seen a range expansion in Britain.  The genome assembly is 421 Mb large and is scaffolded to 33 scaffolds. The genome could not be fully phased. The QV value is 69.4 and the BUSCO gene completeness at 99.0, which indicate a very complete assembly. The contamination screen found minimal contamination and gene annotation identified 17,784 genes.

 Minor comments:

 1) The mitochondrial genome has been assembled, but seems to not be extracted/submitted separately. It would be helpful to have the mt genome submitted separate. It would be beneficial for those that are interested in the mitochondrial genome. 

 2) Please clarify for what analyzes that the abdomen DNA was used for. The material and methods describe DNA isolation from the abdomen, while all of a sudden thorax DNA is used for HiC. This contradiction should be clarified.

 3)The data set used for the blob plot in Figure 2, ERR9248441_cov, is not listed anywhere in table 1. 

 Conclusion: the high quality genome assembly of the common Marbled White Spot offers possibilities to study the range expansion of evolutionary properties of the moth family Noctuidae.

Are sufficient details of methods and materials provided to allow replication by others?

Yes

Is the rationale for creating the dataset(s) clearly described?

Yes

Are the datasets clearly presented in a useable and accessible format?

Yes

Are the protocols appropriate and is the work technically sound?

Yes

Reviewer Expertise:

phylogenomics, transposable elements, mitogenomics

I confirm that I have read this submission and believe that I have an appropriate level of expertise to confirm that it is of an acceptable scientific standard, however I have significant reservations, as outlined above.

# Reviewer report: Reviewer response for version 1

**DOI:** 10.21956/wellcomeopenres.22770.r78587

**Published:** 2024-05-07

**Authors:** Alex Makunin

**Affiliations:** 1 Wellcome Sanger Institute, Hinxton, England, UK

The manuscript by Boyes et al present the chromosomal genome assembly for Protodeltote pygarga moth species. This study is seemingly filling a gap in taxonomic sampling by providing the first reference genome assembly for *Eustrotiinae* subfamily of *Noctuidae* family.

 Overall, the genome note is cohesive and well structured. I can see two minor issues:

 - DNA extraction is described in detail only for abdomen, which seemingly was used for PacBio sequencing, but not for thorax which was used for HI-C.

 - The reasoning for Z chromosome assignment in a homogametic sample is not provided - was it based on size (largest chromosome)?

Are sufficient details of methods and materials provided to allow replication by others?

Yes

Is the rationale for creating the dataset(s) clearly described?

Yes

Are the datasets clearly presented in a useable and accessible format?

Yes

Are the protocols appropriate and is the work technically sound?

Yes

Reviewer Expertise:

comparative genomics

I confirm that I have read this submission and believe that I have an appropriate level of expertise to confirm that it is of an acceptable scientific standard.

# Reviewer report: Reviewer response for version 1

**DOI:** 10.21956/wellcomeopenres.22770.r78593

**Published:** 2024-04-24

**Authors:** Balaji Chattopadhyay

**Affiliations:** 1 Ashoka University, Sonipat, Haryana, India

The authors present a high quality genome assembly of an individual male of the noctuid moth Marbled White spot. The authors performed long-read and Hi-C sequencing and assembly to reconstruct chromosomal pseudomolecules of both nuclear (autosomes and sex chromosomes) and mitochondrial genomes.

 While the overall methodology to generate and analyze the data is robust, I feel that the authors should mention:

 1. details of each method

 2. rationale for using specific methods

 For scientists to follow a similar methodology it is important that the advantages and limitation of the methods are clearly stated. Similarly, for each methods it is also quite important to state the parameters that were used and the reason behind using these particular set of parameters.

Are sufficient details of methods and materials provided to allow replication by others?

Partly

Is the rationale for creating the dataset(s) clearly described?

Yes

Are the datasets clearly presented in a useable and accessible format?

Yes

Are the protocols appropriate and is the work technically sound?

Yes

Reviewer Expertise:

Genomics, Conservation genomics, Biogeography

I confirm that I have read this submission and believe that I have an appropriate level of expertise to confirm that it is of an acceptable scientific standard, however I have significant reservations, as outlined above.
